# Design of Peptide Immunotherapies for MHC Class-II-Associated Autoimmune Disorders

**DOI:** 10.1155/2013/826191

**Published:** 2013-11-13

**Authors:** Masha Fridkis-Hareli

**Affiliations:** ATR LLC, 500 West Boylston Street, Worcester, MA 01606, USA

## Abstract

Autoimmune disorders, that occur when autoreactive immune cells are induced to activate their responses against self-tissues, affect one percent of the world population and represent one of the top 10 leading causes of death. The major histocompatibility complex (MHC) is a principal susceptibility locus for many human autoimmune diseases, in which self-tissue antigens providing targets for pathogenic lymphocytes are bound to HLA molecules encoded by disease-associated alleles. In spite of the attempts to design strategies for inhibition of antigen presentation targeting the MHC-peptide/TCR complex via generation of blocking antibodies, altered peptide ligands (APL), or inhibitors of costimulatory molecules, potent therapies with minimal side effects have yet to be developed. Copaxone (glatiramer acetate, GA) is a random synthetic amino acid copolymer that reduces the relapse rate by about 30% in relapsing-remitting multiple sclerosis (MS) patients. Based on the elucidated binding motifs of Copaxone and of the anchor residues of the immunogenic myelin basic protein (MBP) peptide to HLA-DR molecules, novel copolymers have been designed and proved to be more effective in suppressing MS-like disease in mice. In this report, we describe the rationale for design of second-generation synthetic random copolymers as candidate drugs for a number of MHC class-II-associated autoimmune disorders.

## 1. Introduction

Autoimmunity is a multifactorial process that occurs when autoreactive immune cells are triggered to activate their responses against self-tissues. Autoimmune diseases may affect a single organ or multiple systems of the organism. For example, organ-specific diseases include celiac disease (CD), Hashimoto's thyroiditis, type I diabetes mellitus, multiple sclerosis (MS), myasthenia gravis (MG), and pemphigus vulgaris (PV), whereas systemic diseases include, among others, rheumatoid arthritis (RA) and systemic lupus erythematosus (SLE). The major histocompatibility complex (MHC) on human chromosome 6p21 encodes human leukocyte antigens (HLA), which are cell surface proteins that play a central role in regulation of immune responses through their ability to bind and present processed peptides to T cells [1]. The genetic control of the immune response is mediated by the polymorphic sites within the HLA antigen-binding groove that interact with the bound peptides [1, 2]. 

The MHC is a principal susceptibility locus for many human autoimmune diseases, in which self-tissue antigens, providing targets for pathogenic lymphocytes, are bound and presented by the HLA molecules encoded by susceptibility alleles. The likelihood that early events in disease initiation might be triggered by specific HLA-peptide complexes offers some prospects for therapeutic intervention by design of compounds that interfere with the formation or function of HLA-self-peptide/T cell receptor (TCR) interactions. The ability of MHC class II molecules to bind and present antigenic peptides depends on the amino acid composition of their antigen-binding sites. Amino acid substitutions of the peptide may influence the specificity of the immune response by altering the binding affinity for the MHC class II molecules. Effective inhibition of antigen presentation by disease-associated HLA-DR molecules has been shown in several animal models of autoimmune diseases. Some strategies for inducing immunological tolerance include blocking antigen presentation, supplying altered peptide ligands by routes of intravenous and oral administration or blocking costimulatory molecules [3–6]. 

## 2. Copaxone and the Related Copolymers as Novel Therapies for Autoimmune Diseases

Copolymer 1 (Cop 1, Copaxone, GA) is an immunomodulatory drug approved by FDA in 1997 for relapsing-remitting forms of MS, which reduces the relapse rate by about 30%. It is a random synthetic amino acid copolymer of alanine (A), lysine (K), glutamic acid (E), and tyrosine (Y) in a molar ratio of approximately 5 : 3 : 1.5 : 1 synthesized in solution using N-carboxy-amino acid anhydrides [7]. Initially, this and other related copolymers were used to define the genetic basis of immune responsiveness, now known as class II MHC genes [8, 9]. Later, Cop 1 was found to be effective both in suppression of experimental autoimmune encephalomyelitis (EAE) [10] and in the treatment of relapsing MS [11–13]. Its activity involves, as a first step, binding to class II MHC proteins on the surface of antigen-presenting cells (APC) [14]. Cop 1 was shown to compete with myelin antigens, that is, MBP, proteolipid protein (PLP), or myelin oligodendrocyte glycoprotein (MOG), for activation of specific effector T cells recognizing peptide epitopes derived from these proteins [15, 16] and/or induction of antigen-specific regulatory T cells [17, 18]. Moreover, Cop 1 was shown to bind to class II MHC molecules on APC without prior processing [19] and led to clustering of class II MHC on the surface of APC [20]. 

After completion of phase 3 clinical trials, Cop 1 was approved as a therapy for MS and is currently in wide use. However, as noted above, it reduces the relapse rate by only about 30% and is certainly not curative for the disease. The relationship of therapeutic effectiveness of Cop 1 to the HLA haplotypes of MS patients has been established as more effective in HLA-DR2 (DRB1*1501)-positive, to which the disease is linked, than in HLA-DR2-negative patients [21]. Similarly, the ability of Cop 1 to inhibit HLA-DR2-restricted T cell clones derived from MS patients has been reported [22]. Virtually, all of the large variety of copolymers found in the random mixture of YEAK bound to purified human HLA-DR1, -DR2, and -DR4 molecules, showing that Cop 1 did indeed bind to purified class II MHC proteins. It also competed for binding of the immunodominant epitope of MBP 85-99 to HLA-DR2 (DRB1*1501) and inhibited responses of DR2-restricted T cells to this epitope [22, 23]. In order to identify the binding motifs of Cop 1 bound to the HLA-DR molecules, pool sequencing of Cop 1-bound fragments eluted from HLA-DR1, -DR2, and -DR4 proteins has been performed [24]. In this elegant study, the protruding N-terminal ends of Cop 1 bound to recombinant empty HLA-DR1, -DR2, or -DR4 molecules, expressed in insect cells, were treated with aminopeptidase I, followed by elution, HPLC separation, and pool sequencing. In contrast to the untreated or unbound copolymer, which showed similar patterns of the amino acid composition and sequencing, the digested material exhibited distinct motifs at some positions within the binding site. Thus, there were increases in the levels of E at the first and second cycles, of K at the second and third cycles (corresponding to the P-1 position stabilizing the interactions of the bound peptide with the T cell receptor), and of Y (presumably at P1 of the bound peptide) at the third to fifth cycles, regardless of the HLA-DR molecule employed. No preference was seen at the following cycles that were mainly represented by A [24]. These pooled HLA-DR binding epitopes provided clues to the components of Cop 1 that are biologically active in suppressing MS and possibly RA.

Thus, further investigation of the mechanisms involved, as well as examination of additional copolymers of this type, has been carried out with the goal of developing improved therapeutic agents for MS. Novel peptides and copolymers have been designed on the basis of the binding motifs of Cop 1 and the autoantigenic peptides specific for MS and RA. In RA studies, peptides containing Y at P1 of the binding site were particularly effective as inhibitors of the binding of collagen type II p261-273 epitope (a candidate autoantigen in RA) to HLA-DR1 or -DR4 proteins. Moreover, several of the synthetic peptides were also potent inhibitors of the p261-273-reactive T cell clones [25]. In a different RA study, Cop 1-related copolymers inhibited both the binding of p261-273 to RA-associated HLA-DR1 and -DR4 molecules and the response of DR1- and DR4-restricted p261-273-specific T cell clones [26]. On the other hand, in MS-related studies, peptides synthesized according to the binding motifs of Cop 1 and of the immunodominant epitope of MBP p85-99 differentially inhibited binding of these antigens to disease-associated HLA-DR2 (DRB1*1501) molecules. In particular, two peptides with residue K at position P-1 inhibited effectively the binding of both MBP p85-99 and GA to the HLA-DR2 molecules as well as decreased IL-2 production by the two MBP-specific HLA-DR2-restricted T cell clones [22]. High affinity modified synthetic peptide 15-mers inhibited even more strongly both the binding of MBP 85-99 to HLA-DR2 and IL-2 production by the two MBP 85-99-specific HLA-DR2-restricted T cells [27]. These peptides also suppressed EAE induced by MBP 85-99 in humanized mice, and PLP 139-151-induced EAE in SJL/J mice. Moreover, none of these peptide inhibitors cross-reacted with MBP 85-99- or PLP 139-151-specific T cells. In both cases, spleen and lymph node cultures stimulated with these peptides produced large amounts of Th2 cytokines (IL-4 and IL-10), and adoptive transfer of established T cell lines suppressed disease induction [28].

The effects and the mechanisms of Cop 1-related copolymers composed of three or four amino acids have been examined in the EAE model. In the earlier study, the binding of copolymers to purified class II MHC molecules with differential affinities has been reported [29]. The three amino acid copolymer YAK bound to purified human HLA-DR1 or -DR4 molecules with affinity higher than YEA, EAK, or YEK, whereas EAK was the better binder of HLA-DR2 molecules. On the other hand, YEA and YAK inhibited the binding of biotinylated GA to these molecules 10-fold more efficiently than YEK. Finally, YEA, YAK, and EAK were cross-reactive with GA at the level of GA-specific T cell lines and clones of mouse or human origin [29]. In the subsequent study, 14-, 35-, and 50-mers of random sequences composed of four amino acids at certain ratios have been synthesized as described above [30]. These copolymers were examined in three ways: (a) binding to “empty” HLA-DR2 (DRB1*1501) synthesized in a baculovirus system, (b) inhibition of the four MBP 85-99- or PLP 40-60-specific HLA-DR2-restricted T cell clones, as well as the two PLP 139-151-specific H-2^s^-restricted T cell hybridomas, and finally (c) ability to suppress EAE induced by either PLP 139-151 or whole spinal cord homogenate (WSCH) in H-2^s^ mice. The following results were obtained. (a) In the binding experiment, VEAK and FEAK were less effective than Copaxone (commercial batch) or YEAK (custom synthesized) in competing for binding of biotinylated MBP 86-100; (b) when tested as inhibitors of MBP 85-99- or PLP 40-60-induced stimulation of the four HLA-DR2-restricted T cell clones or hybridomas, VEAK was substantially less effective than Copaxone or YEAK in inhibition of proliferation, while FEAK was equivalent to Copaxone using two of these clones and slightly less effective in the other two cases. Using the two PLP 139-151-specific H-2^s^-restricted T cell hybridomas, again FEAK was approximately equivalent to Copaxone or YEAK, while VEAK was half as effective. In both the binding and the inhibition of T cell proliferation experiments, the 50-mers of all of the copolymers used were much more effective than the 35-mers or the 14-mers; (c) VEAK and Copaxone were equally effective in partially reducing the severity of EAE induced by PLP 139-151 in H-2^s^ mice, while FEAK completely prevented the appearance of disease, except for a few mice which developed a transient +1 score (limp tail). In immunohistology, brain sections of control animals and VEAK-treated animals showed substantial demyelination, while animals treated with Copaxone, YEAK, or FEAK were completely normal. WSCH was also used to induce the disease, in which case a milder disease was produced, perhaps more comparable to MS. The mild disease persisted in some animals treated with either GA or YEAK and in an even larger number of animals treated with VEAK. However, no disease was detected in any animals treated with FEAK [30].

**Figure 1:**
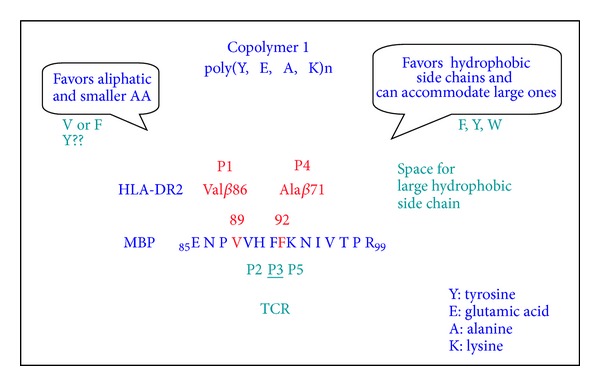
Schematic representation of the rational for design of the novel copolymer YFAK.

Based on the above observations, additional copolymers have been designed as 50-mers. In this group, F was substituted with E because (a) E seemed unnecessary. Moreover, the P1 pocket of DRB1*1501 includes *β*86Val, resulting in a small pocket that can accommodate F but for which Y is too large to be accommodated; (b) the residue occurring at P4 in MBP 85-99 is F, although Y would provide a better fit.  Figure 1 depicts the rational for design of the novel copolymer YFAK. To determine whether novel copolymers competed with the autoantigenic MS-associated epitope MBP 85-99 for binding to HLA-DR2, competitive binding assays were carried out with biotinylated MBP 86-100 and unlabeled random copolymers. All of the YFAK and FAK 50-mers were equally effective and equivalent to or better than any other copolymer or Copaxone in binding to HLA-DR2. Moreover, YFAK and FAK were much more effective than Copaxone in inhibition of MBP 85-99-specific HLA-DR2-restricted T cell clones. Most importantly, these novel copolymers YFAK and FAK were much more effective than Copaxone in suppression of EAE induced in the susceptible SJL/J (H-2^s^) mouse strain. None of the animals treated with YFAK developed a significant disease. Furthermore, these copolymers have been shown to shift the immune responses of autoantigen-specific T cells from Th1 to Th2 phenotype, with IL-4 and IL-10-characteristic cytokine profiles [28]. In the latter study, the enhanced efficacy of these copolymers in EAE induced in SJL/J mice with PLP 139-151 epitope was demonstrated by using three protocols: (i) simultaneous administration of autoantigen and copolymer (termed prevention), (ii) pretreatment with copolymers (vaccination), or (iii) administration of copolymers after disease onset (treatment). Strikingly, in the treatment protocol administration of soluble VWAK and YFAK after onset of disease led to stasis of its progression and suppression of histopathological evidence of EAE. In all of these protocols, the copolymers showed a pronounced suppressive effect on PLP 139-151-induced EAE in the order VWAK > YFAK ≫ Copaxone.

The mechanisms by which these effects are achieved have been examined in several types of assays: binding of copolymers to I-A^s^ in competition with PLP 139-151 (blocking), cytokine production by T cells (T helper 2 polarization), and transfer of protection by CD3^+^ splenocytes or, notably, by copolymer-specific T cell lines (induction of regulatory T cells). Importantly, these copolymers were shown to bind to I-A^s^, the only class II MHC protein expressed in SJL/J mice and to cluster and compete with PLP 139-151 for binding to I-A^s^. Previously, aggregates (clusters) of I-A^s^ molecules after Cop 1 binding were detected on the surface of antigen-presenting cells from SJL/J mice [20]. YFAK and VWAK were more potent than Copaxone in binding to mouse I-A^s^ molecules and in competing for PLP 139-151 binding. In addition to the competitive binding potential, the copolymers were also inhibitors of the expansion of PLP 139-151-specific T cells, both *in vitro* and *in vivo*, again in the order VWAK > YFAK > GA.

The generation of copolymer-specific regulatory T cells that secrete IL-4 and IL-10 and are independent of the immunizing autoantigen is very prominent among the multiple mechanisms that account for the observed suppressive effect of copolymers in EAE. Furthermore, copolymers shifted the T cell immune response from a classical Th1 phenotype toward a Th2 response (immune deviation). In SJL/J mice, restimulation of splenocytes from PLP 139-151-immunized animals with PLP 139-151 *in vitro* induced the production of IFN-*γ* but not IL-4 or IL-10. However, splenocytes from mice coimmunized with PLP 139-151, and copolymers when restimulated with their corresponding copolymers also produced IL-4 and IL-10 without much alteration in the production of IFN-*γ*. These cytokines may be produced by copolymer-specific T cells with a negligible contribution, if any, from PLP 139-151-reactive T cells. Third, copolymers may mediate their effects by inducing copolymer-specific T cells with the Th2 phenotype. The copolymers upon immunization of SJL/J mice induced a copolymer-specific T cell response; that is, the copolymers are immunogenic. Moreover, adoptive transfer of copolymer-specific T cells reduced markedly the severity of EAE, suggesting that they produce Th2 cytokines without copolymer restimulation. Of note is that the copolymer-specific T cell lines are antigen nonspecific; that is, they can be generated and they respond to copolymers in the absence of antigen. Thus, they may be useful in the treatment of other autoimmune diseases or in those where several autoantigens are involved, as is likely to be the case in MS.

However, whatever the mechanism, the first step must be binding to a class II MHC protein. The copolymers were optimized for binding to HLA-DR2 but they are likely to bind promiscuously to class II MHC proteins, including I-A^s^, with varying affinities. A number of mechanisms, in addition to blocking and immune deviation, resulting from the generation of copolymer-specific T cells, such as TCR competition or induction of anergy, may be operative. Induction of hyporesponsive T cells (anergy) in MS patients after continuous administration of GA has been observed. The generation of copolymer-specific CD4^+^ Th2 cell lines that secrete IL-4 and IL-10 and can adoptively transfer resistance to EAE appears very prominent among these mechanisms. Copolymers might also suppress disease through modulating CNS antigen-presenting cells, that is, microglia.

Different copolymers may have different mechanisms of suppression. VWAK appeared to be less able to generate T cell lines and it also generates larger amounts of IL-4 and lower amounts of IFN-*γ*, yet it suppressed EAE somewhat more effectively. However, VWAK bound more tightly to I-A^s^ and thus might be a better blocking agent. YFAK was much more effective in stimulating copolymer-specific T cell lines and production of anti-inflammatory cytokines IL-4 and IL-10 and thus should be much more effective in disease reduction if immune deviation is the mechanism. Conversely, VWAK induced IL-4 and IL-10 production only relatively weakly and, like YFAK, also induced T cell anergy relatively poorly [28]. Thus, a combination of mechanisms may be involved in the reduction of severity of EAE. Altogether, these data suggested that random synthetic copolymers designed according to the binding motifs of the human immunodominant epitope MBP 85-99 and the binding pockets of HLA-DR2 might be more beneficial than GA in treatment of MS.

## 3. Mechanism of Action 

The exact mechanism of activity of the random copolymers remains to be elucidated. From the *in vitro* studies, using EBV-transformed B cell lines of MS patients and healthy donors and purified HLA-DR proteins, it follows that Cop 1 and the related copolymers bind promiscuously to various HLA-DR alleles and inhibit antigen-specific T cell responses [14, 23], suggesting that the copolymers may act as altered peptide ligands. Moreover, in EAE (the animal model of MS) the copolymers have been shown to shift the immune responses of autoantigen-specific T cells from Th1 to Th2 phenotype, with IL-4 and IL-10-characteristic cytokine profiles [28]. The mechanism of activity may also involve the induction of copolymer-specific Th2 cells, which inhibit manifestations of EAE by secretion of cytokines or by yet another pathway. Recently, the role of copolymers in suppression of autoimmune inflammation was expanded to involve antigen-presenting cells and to promote regulatory B cell properties [31]. In this regard, the role of both the innate and the adaptive immunity has been proposed in two reports showing secretion of macrophage-specific chemokines upon administration of copolymers to mice in the absence of MHC class II receptors [32, 33]. Recently, interactions between the copolymers and the APC have been further characterized suggesting that these interactions are charge dependent [34].

Thus, several mechanisms, including ionic interactions, binding to MHC class II proteins, blocking and immune deviation, T cell receptor competition, or induction of anergy, may be operative in the process of inhibition of EAE by the copolymers. It is possible that similar mechanisms would apply to the effects of the copolymers on a number of other autoimmune diseases. For example, random copolymers have been shown to inhibit experimental autoimmune uveitis in mice by induction of immunosuppression and secretion of Th2 cytokines [35]. 

## 4. Candidate Autoimmune Diseases as Targets for Copolymer-Based Drug Design

### 4.1. Celiac Disease

Celiac disease is an inflammatory disorder of the small intestine caused by an immune response to ingested wheat gluten and similar proteins of rye and barley. It affects at least 1 in 200 individuals, corresponding to roughly three million patients in Western Europe and Northern America alone [36]. Data accumulated since the discovery of gluten specific T cells in the intestine of CD patients in the early 1990s have allowed the deciphering of the interplay between the triggering environmental factor, gluten, the main genetic risk factor, the HLA-DQ2/8 haplotypes, and the autoantigen, the enzyme tissue transglutaminase (tTG). These findings established a key role of adaptive immunity orchestrated by lamina propria T cells responding to a set of gluten derived peptides. The HLA-DQA1*05 and HLA-DQB1*02 genes, which are carried by most CD patients, encode HLA-DQ2, while a minority of the patients carry HLA-DQ8 gene products. These proteins bind and present peptide fragments of gluten proteins to T lymphocytes present in the celiac mucosal lesion, leading to their activation, which in turn initiates a cascade of events that eventually leads to villous atrophy and crypt cell hyperplasia [37, 38]. 

### 4.2. Current Treatments for Celiac Disease

Glucocorticoids and dietary restrictions are the only current treatments for CD. Hence, there is a need for novel drugs with no side effects of the present therapies, and which adequately address the innate heterogeneity of the immune system. Agents that bind selectively to HLA alleles associated with CD, and thus interfering with the ability of gluten peptides to bind, which would lead to inactivation of autoreactive T cells in the mucosa, could be potentially effective in the treatment of CD. 

### 4.3. Rheumatoid Arthritis

Rheumatoid arthritis (RA) is one of the most common forms of arthritis affecting an estimated 9.7 million people worldwide (approximately 3 million in the United States and Canada). RA affects twice as many women as men between the ages of 25 and 50; however, it can also appear in other age groups. RA is an autoimmune disease characterized by persistent inflammatory synovitis leading to various degrees of cartilage destruction, bone erosion, and ultimately joint deformity and loss of joint function [39]. Although the etiology of RA is unknown, it is well established that inherited susceptibility to RA is associated with the genes encoding the human class II MHC molecules HLA-DR4 and -DR1 [40]. The serologically defined HLA-DR4 group is divided into at least 19 subtypes of which DRB1*0401 and DRB1*0404 are the predominant alleles found in approximately 80% Caucasian RA patients, while DRB1*0405 predisposes to RA in Japanese. In other racial groups, such as Israeli Jews, DRB1*0101 accounts for susceptibility to RA [40]. Of patients with a particularly severe form of arthritis (Felty's syndrome), 95% express HLA-DR4 molecule, with increased disease frequency mainly due to the presence of DRB1*0401 [41]. The disease-associated molecules share sequences at positions 67–71 of the DR*β* chain [42–44] which is found in the peptide-binding site of the class II molecules [45, 46] and known as a “shared epitope.” The majority of RA patients in the groups that do not carry the HLA-DR4 subtype, although they carry alleles with amino acid variations in the peptide-binding site, share identical residues with the DRB1*0401 sequence at the shared epitope. 

The shared epitope residues are critical in selecting specific amino acids at position P4 of peptides that will bind to DR4. In particular, among peptides with electrostatically charged residues at P4, only those with negatively charged residues (Asp and Glu) at this position bind to DR molecules with associated increased susceptibility to RA. Peptides with such residues do not bind to molecules such as DRB1*0402, an allotype that differs in sequence only at the shared epitope and that is not associated with increased susceptibility to the disease [47, 48]. The sequence differences between DRB1*0401 and DRB1*0402 change the electrostatic charge in the pocket that binds the P4 side chain of peptides. Residue DR*β*71 is positively charged (Lys or Arg) in DRB1*0401 and is negatively charged in the nonassociated DRB1*0402. This suggests that the mechanism by which specific DR molecules are associated with RA could involve their selectivity for binding a peptide with the negatively charged P4 [47]. Such peptide selectivity may alter the T cell repertoire during T cell differentiation in the thymus or selectively activate arthritogenic T cells at the level of antigen presentation, leading to the development of autoimmunity [49]. The antigens responsible for the induction of RA are unknown, but type II collagen (CII) is a candidate autoantigen since it is the predominant protein of joint cartilage and since autoantibodies against CII are found in elevated levels in the serum and joints of patients with RA [50]. RA can be induced by immunization with CII [51, 52]. The crystal structure of HLA-DR4 complexed with a peptide from human collagen type II has been resolved, allowing better understanding of the structural basis for the selective binding of peptides to RA-associated HLA-DR4 molecules [53]. 

### 4.4. Current Treatments for RA

There is no cure or prevention for RA today. A variety of treatments exist to treat the symptoms resulting in less pain, stiffness, and easier movement. Four major treatment approaches are recognized in the management of RA: medicine (pharmacological), physical (exercise), joint protection and lifestyle changes, and surgery. There are four types of medicine used to treat RA: (1) nonsteroidal anti-inflammatory drugs (NSAIDs) reduce pain when taken at a low dose, and relieve inflammation when taken at a higher dose; however, they do not prevent further joint damage. Common NSAIDs include Aspirin, Anacin, Advil, and Motrin IB, which can be purchased without a prescription. Other NSAIDs that require a prescription include Naprosyn, Relafen, Indocid, Voltaren, Feldene, and Clinoril. Taking more than one NSAID at a time increases the possibility of side effects, particularly stomach problems such as heartburn, ulcers, and bleeding. (2) Disease-modifying antirheumatic drugs (DMARDs) target the cells of the immune system causing the inflammation but do not reverse permanent joint damage. It takes about two to six months before they begin to reduce pain and swelling. The most common medications are gold salts, methotrexate, sulfasalazine, hydroxychloroquine, chloroquine, and azathioprine. A DMARD is usually prescribed in addition to an NSAID or prednisone. Side effects may include mouth sores, diarrhea, and nausea. More serious side effects, monitored through regular blood and urine tests, include liver damage, excessive lowering of white blood cell count (increasing susceptibility to certain infections), and platelet count (affecting blood clotting). (3) Corticosteroids are used to treat extreme inflammation that is accompanied by severe pain and stiffness. The most common form is oral prednisone. Side effects from long term use may include cataracts, high blood pressure, sleep problems, muscle loss, bruising, thinning of the bones (osteoporosis), weight gain, and susceptibility to infections. Corticosteroids sometimes are given as injection into one or more joints or other areas of inflammation. While eliminating the serious side effects, injections may have their own harmful results on the joints if given more than a few times a year. (4) Biological response modifiers (BRMs) are generally indicated for people with aggressive, debilitating arthritis, who have not responded to one or more DMARDs. The BRMs are designed to target the proinflammatory cytokines, which contribute to the disease process. There are currently three approved BRMs: etanercept (brand name: Enbrel), infliximab (brand name: Remicade), and Anakinra (brand name: Kineret). It has been shown that some patients treated with Enbrel develop tuberculosis.

### 4.5. Type I Diabetes Mellitus

Diabetes mellitus (DM) is a leading cause of morbidity and mortality in the United States, with the incidence increasing worldwide. Scandinavia has the highest rate for type I DM (35/100,000 per year), intermediate for Europe and Northern America (17/100,000 per year), and lower for Pacific Rim (3/100,000). Complications of DM are severe and include renal failure, cardiovascular disease, blindness, and amputations [54]. Type I diabetes results from autoimmune pancreatic *β*-cell distraction, which leads to insulin deficiency. Pancreatic components which are targeted by autoimmune process include insulin, glutamic acid decarboxylase (GAD), ICA-512/IA-2 (homology with tyrosine phosphatase), and phogrin (insulin secretary granule protein). Most individuals with type I DM carry the HLA-DR3 and/or DR4 haplotype. The strongest associations (40% of children) are with DQA1*0301, DQB1*0302, DQA1*501, and DQB1*0201. In contrast, DQA1*0102 and DQB1*0602 are protective alleles [55, 56].

### 4.6. Therapies for Type I DM

Treatments for type I DM include injections of insulin, immunosuppressants, and glucocorticoids, which may cause severe side effects. The only cure for type 1 diabetes is a pancreas transplant, which is rarely done. Because both pancreas transplants and kidney transplants require lifelong use of powerful drugs to suppress immune reactions that can reject the organs, pancreatic transplants are usually done in those with type 1 diabetes who also need a kidney transplant. The side effects of immune-suppressive drugs can be severe and even worse than the disease. One or two people out of every 10 who get the surgery die within a year. And the new pancreas is rejected by half of the people who get this operation. If the transplant fails, diabetes returns. Other drugs include recently approved Symlin (Pramlintide Acetate), as an adjunct treatment in patients who use mealtime insulin therapy and who have failed to achieve desired glucose control despite optimal insulin therapy, and Levemir (Insulin Detemir) which is indicated for once or twice daily subcutaneous administration in the treatment of adult patients with Type 1 DM who require basal (long-acting) insulin for the control of hyperglycemia.

## 5. Design of Copolymer-Based Candidate Therapies for Autoimmune Diseases

Up to date, random synthetic copolymers have been designed based on the concept of combining the information on the binding motifs of Copaxone and the structure of the immunodominant peptide of the autoantigen specific for an autoimmune disease. Several considerations have to be taken into account when using this approach. (A) The candidate autoantigens leading to the induction of autoimmunity in humans have partially been identified, and thus, in many cases, their amino acid sequence is unavailable. (B) There might be several autoantigenic peptides, which derive from the same or different self-proteins, containing distinct amino acid sequences. Consequently, it would be challenging to design a copolymer based on the structure of several peptide antigens bound to the same class II molecule. (C) Autoimmune disease might be triggered by factors other than binding of the autoantigenic peptide to the MHC protein in the susceptible individual (e.g., viral peptide of the unknown sequence might initiate events leading to disease induction). Therefore, in this case, autoantigenic peptide-based design approach is irrelevant. (D) Autoantigenic peptides might undergo structural modifications in the course of the disease. These include enzymatic as well as chemical changes. (E) Autoantigenic peptides bind class II MHC molecules with relatively low affinity; therefore, approach of competitive inhibition using the candidate drug composition of the amino acids interacting weakly with the pockets of the MHC might not be efficient.

Novel compounds have been designed using a different approach from the one described above, namely, taking into consideration the random structure of Copaxone and the epitope binding sites of the MHC class II proteins having strong association with a number of autoimmune diseases, including celiac disease, rheumatoid arthritis, type I diabetes, and pemphigus vulgaris. For all of these diseases, the detailed structure of HLA molecules along with the amino acid residues lining the binding groove, and the crystal structure of the disease-associated epitope bound to the HLA protein, have been well established [53, 57]. Similar to the beneficial effects of Copaxone in relapsing-remitting MS, these novel compositions are believed to have promising (efficacious and safe) therapeutic profiles in autoimmune diseases for which they have been designed. Studies to test their functional activity in animal models of autoimmunity are currently ongoing. 

## 6. Conclusions

More effective therapies with tolerable side effects are needed to treat patients afflicted with autoimmune diseases. Clinical trials of therapeutic peptides have been conducted in a range of autoimmune disorders, including DM, RA, SLE, and MS, with various routes of administration and dosing schedules being investigated (reviewed in [58] and on www.clinicaltrials.gov). Copaxone, a blockbuster drug for relapsing-remitting MS, has been evaluated for a number of neurodegenerative disorders. Furthermore, a family of novel synthetic copolymers, designed on the basis of the immunological properties of Copaxone, and specifically, YFAK, is currently in pharmaceutical development for MS. It is of interest to examine additional copolymers as candidate treatments for MHC class-II-associated autoimmune disorders due to their immunomodulatory and anti-inflammatory activities. These compounds are believed to provide patients with safe, long-lasting, and substantial improvements in quality of life.
